# Next steps for the East, Central and Southern Africa College of Family Physicians (ECSA-CFP)

**DOI:** 10.4102/phcfm.v16i1.4753

**Published:** 2024-10-31

**Authors:** Innocent K. Besigye, Mpundu Makasa, Martha Makwero, Jacob S. Shabani, Sunanda Ray

**Affiliations:** 1 Department of Family Medicine, College of Health Sciences, Makerere University, Kampala, Uganda; 2 Department of Community and Family Medicine, School of Public Health, University of Zambia, Lusaka, Zambia; 3 Department of Family Medicine, College of Health Sciences, Kamuzu University, Blantyre, Malawi; 4 Department of Family Medicine, Faculty of Health Sciences, Aga Khan University, Nairobi, Kenya; 5 Department of Medical Education Unit, Faculty of Medicine, University of Botswana, Gaborone, Botswana

The College of Family Physicians of East, Central and Southern Africa (ECSA-CFP) was recently approved by the ECSA Health Community and endorsed by the 73rd Ministers of Health Conference at Arusha, 20–21 June 2024. The College aims to significantly increase the number of family physicians (FPs) in the ECSA region, who will lead developments in Family Medicine (FM), primary and district healthcare (see Box 1). The College’s training model relies on distributed learning platforms based at district hospitals, with supervision, mentorship and assessment by faculty at medical schools and teaching hospitals. Resources for such programmes, especially for assessment and quality assurance of training, are scarce in the region. There are few FM educators skilled in designing distributed learning modules and assessment plans for trainees. The College’s primary focus will therefore be on capacity-building: supporting existing MMed FM programmes in training, assessment and examinations, and thereafter to establish the College’s competency-based training and licensing programme that will be recognised by the regulatory authorities of member countries.

BOX 1 Vision and mission of East, Central and Southern Africa College of Family Physicians.

**Vision of ECSA-CFP**

To lead the transformation of primary healthcare in the ECSA region and strengthening the health workforce by providing high quality education, training, research and advocacy in Family Medicine.

**Mission:**

- to increase the availability of specialist family physicians in the ECSA region through high quality postgraduate training and assessment
- to enable collaboration between existing postgraduate Family Medicine training programmes and the sharing of expertise in both training and assessment
- to improve healthcare outcomes for individuals, families and communities through continuous, comprehensive and person-centred healthcare.

*Source:* The constitution of the College of Family Physicians Eastern, Central and Southern Africa (ECSA CFP)

ECSA-CFP, East, Central and Southern Africa College of Family Physicians.

The College was initiated by a partnership of FPs from several ECSA countries, committed to improving FM education in the region. Following publication of a proposal to establish the College,^1^ the ECSA-CFP concept was endorsed by participants at the Primary Care and Family Medicine (Primafamed) Network Conferences in Malawi (2022) and Johannesburg (2023). A steering group developed and submitted the constitution, curriculum, strategic plan and roadmap to the ECSA College of Health Sciences, which approved them in June 2024. An Executive Committee of key officials was subsequently ratified by a council of country representatives, with three sub-committees tasked with accreditation, training, finance and membership.

## Priority areas

The new ECSA-CFP leadership have identified the following priority areas:

- Setting up administrative and operational structures, creating a register of Fellows and defining accreditation standards. National FP associations will manage recruitment of Fellows and collection of membership fees.
- Pooling academic resources and expertise to support existing university FM specialist training programmes (MMed), with their training, assessments and examinations.
- Adapting the format of the single College curriculum by national FP associations to meet each country’s regulatory standards.
- Developing a regional competency-based training programme and licencing examination that is recognised by the regulatory authorities of member countries.
- Developing regional and international partnerships to secure funding for face-to-face and online meetings, MMed exchange visits, external examiner visits, cross-border supervision of trainees, regional courses, and training-of-trainer workshops.
- Building strong partnerships with national health ministries, academic institutions, international health organisations, non-governmental organisations, diaspora and private sector stakeholders to support the College’s initiatives, facilitate knowledge exchange and sharing of best practices.
- Showcasing the benefits of enhanced quality of primary and district hospital care through collaborative research, publications, workshops and conferences, including developing research supervisory capacity within training programmes.
- Leveraging digital technology and E-learning platforms to build on Primafamed’s successful online workshops, to create flexible training opportunities for FPs, including faculty development, particularly in remote areas.
- Establishing a robust monitoring and evaluation framework to help the College track progress, identify challenges and make necessary adjustments.

## Competency-based family physician training and assessment

The College training will emphasise the roles of FPs as care providers, consultants, capacity builders, clinical trainers, clinical governance leaders, and champions of community-oriented primary care.^2^ The curriculum includes clinical skills as well as competencies in leadership, management, training and research to prepare FPs to take on multifaceted roles within the healthcare system. Retaining trained FPs within the region will be prioritised by creating a supportive professional environment with continuous education opportunities, competitive remuneration, and career advancement pathways. Establishing a network of FPs across the region through the College will foster peer support and collaboration, enhancing professional satisfaction and reducing the risk of low morale and burnout. The College will build capacity for teaching and assessments of FPs, benchmarking quality standards across the region. Educational campaigns highlighting the benefits of having FPs can create more public demand for such expertise in primary care and hospitals. Working in partnership with public health colleagues, the College will enhance community-oriented primary care, as well as promoting joint responses to disease outbreaks, monitoring health trends and the impact of climate change on health.

For the College to achieve its objectives, there must be alignment with national and regional health policies and priorities. This includes recognising FPs as essential components of the healthcare workforce, including them in policy development, and providing adequate funding for their training and deployment. Policy alignment also involves addressing regulatory and legislative frameworks to ensure that FPs can practise to the full extent of their training, enhancing the role of FPs in primary care and district hospital service delivery, and ensuring that they are included in decision-making processes at all health system levels.

## Conclusion

The approval of the College is a transformative step for healthcare in East, Central, and Southern Africa. Moving forward, the focus will be on effective implementation, capacity building, enhanced partnerships, policy alignment, leveraging technology, and monitoring and evaluation. By addressing these areas, the ECSA-CFP can fulfil its vision of strengthening FM and primary healthcare and improving health outcomes for the people of the region.

The authors are grateful to Prof. Bob Mash and the Primafamed Network for their unwavering support in establishing the ECSA-CFP.
